# Diabetic Ketoacidosis at Diagnosis is Associated With Adverse Long‐Term Glycaemic Trajectories and Retinopathy in Adult‐Onset Type 1 Diabetes. A Single‐Centre Retrospective Cohort Study

**DOI:** 10.1002/dmrr.70249

**Published:** 2026-11-03

**Authors:** Riccardo Maria Pollis, Federico Boscari, Damiano Furlanetto, Cristina Bittante, Maria Cristina Crepaldi, Monica Vedovato, Daniela Bruttomesso, Alberto Maran, Benedetta Maria Bonora, Mario Luca Morieri, Gian Paolo Fadini

**Affiliations:** ^1^ Department of Medicine University of Padova Padua Italy; ^2^ Veneto Institute of Molecular Medicine Padua Italy

**Keywords:** adulthood, autoimmune, comorbidities, observational

## Abstract

**Aims:**

The long‐term significance of diabetic ketoacidosis (DKA) at the diagnosis of adult‐onset type 1 diabetes (T1D) is poorly understood. We investigated the association between DKA at diagnosis and long‐term outcomes.

**Methods:**

In this retrospective study, we analysed adults with T1D diagnosed at age ≥ 18 years who attended a single tertiary diabetes centre between 2000 and 2025. Participants were classified according to the presence or absence of DKA at diagnosis. Longitudinal changes in HbA1c were analysed using mixed models. Incident chronic complications were evaluated using Cox regression models.

**Results:**

Among 517 adults with T1D (47.2% women), 112 (21.7%) presented with DKA at diagnosis. The frequency of DKA at diagnosis increased from 13.2% in 2000–2008 to 34.6% after 2016 (*p* < 0.001). During a median (IQR) follow‐up of 9.3 (3.5–15.5) years after cohort entry, individuals presenting with DKA had persistently higher HbA1c levels than those without DKA, with an adjusted between‐group difference of 0.37% (95% CI 0.20, 0.53; *p* < 0.001). When data were aligned to the diabetes onset date, the estimated mean difference was 0.66% (*p* < 0.001). In exploratory analyses, DKA at diagnosis was associated with a higher incidence of retinopathy (HR 1.61; 95% CI 1.02–2.55; *p* = 0.041). The association was confirmed after accounting for delayed cohort entry and was attenuated by adjusting for mean HbA1c during follow‐up.

**Conclusions:**

Among individuals with adult‐onset T1D attending a tertiary diabetes clinic, DKA at diagnosis was associated with persistently less favourable glycaemic control during long‐term follow‐up. Interpretation is limited by the single‐centre retrospective design and delayed cohort entry.

## 1 Introduction

Type 1 diabetes (T1D) can present with a broad spectrum of clinical manifestations, ranging from incidental hyperglycemia to severe diabetic ketoacidosis (DKA). DKA remains the presenting feature of T1D in a substantial proportion of newly diagnosed individuals [1]. Despite improvements in awareness, the frequency of DKA at diagnosis appears to be increasing in several populations [2, 3].

In children and adolescents, presentation with DKA has been associated with more severe metabolic decompensation, lower beta‐cell function, poorer glycaemic control, and less favourable long‐term outcomes [4, 5]. These observations suggest that DKA may be a manifestation of delayed diagnosis and identifies individuals with a more aggressive disease phenotype characterised by rapid beta‐cell destruction, greater metabolic instability, and/or unfavourable socioeconomic determinants of health [6].

Whether similar considerations apply to adult‐onset T1D is poorly understood [7]. Although around half of new T1D cases occur during adulthood [8], most studies investigating the clinical implications of DKA at diagnosis have focused on paediatric populations. Adult‐onset T1D is clinically heterogeneous [9] and often characterised by variable beta‐cell decline, differences in diagnostic delay, and distinct patterns of healthcare utilisation, making the prognostic significance of DKA at diagnosis uncertain [8, 10]. Filling such gaps would reinforce the clinical and public health importance of preventing DKA at disease onset [2, 11, 12], beyond the immediate risks of acute metabolic decompensation.

Here, we evaluated the association between DKA at diagnosis of adult‐onset T1D and long‐term metabolic outcomes and complication burden in a large retrospective cohort followed for several decades. We also explored temporal trends in the frequency of DKA at diagnosis and compared outcomes according to acute versus non‐acute disease presentation.

## 2 Methods

### 2.1 Study Design and Objectives

This was a single‐centre retrospective longitudinal study. The study was part of the DiOR protocol (Diabetes Outcome Research) approved by the Territorial Ethics Committee for the Central‐Eastern Veneto Area (CET‐ACEV, prot. *n*° 422n/AO/23). All participants provided written informed consent for the reuse of data collected in their electronic medical records for the scope of the project.

The main objective of the study was to detect differences in the long‐term HbA1c trajectory and complications of adult‐onset T1D between those who had DKA at diagnosis and those who had not. The secondary objective was to compare acute versus non‐acute onset, irrespective of DKA.

### 2.2 Participants

Individuals with T1D were retrospectively identified in the database of the Diabetes Outpatient Clinic of the University Hospital of Padova based on the diagnosis coded in the system. The diagnosis of T1D was manually reviewed according to the AABBCC approach (Age, Autoimmunity, Body habitus, Background, Control, Comorbidities) [13], integrating clinical phenotype and the available biochemical and immunological information. Islet autoantibody testing was not uniform over the study period, reflecting changes in routine clinical practice and assay availability over more than two decades. Earlier assessments mainly included ICA, GADA and IA‐2A, whereas ZnT8A and other assays became available more recently. Autoantibody positivity was not mandatory for inclusion, and antibody‐negative cases were strictly adjudicated on the basis of the clinical phenotype and available diagnostic information. C‐peptide was considered when available but was not systematically required for diagnostic classification. Only those with onset at 18 years of age or older were retained in the present analysis. There were no other inclusion/exclusion criteria.

### 2.3 Exposures and Endpoints

The primary exposure was DKA at diagnosis. Onset with DKA was defined when individuals received a diagnosis of T1D as a consequence of an episode of ketoacidosis. Information used to define DKA was reviewed manually by a diabetes expert blinded to study outcomes to confirm the diagnosis according to ongoing standards [1, 14]. Cases with uncertain classification were further reviewed and adjudicated through discussion with two additional authors until consensus was reached. For the primary analysis, participants were divided in two groups: with or without DKA at diagnosis.

The secondary exposure of interest was acute onset. Acute vs. non‐acute onset was retrospectively reconstructed from the narrative description of disease presentation in the clinical records. T1D onset was defined as acute when T1D was diagnosed as a consequence of an emergency visit or hospitalisation for typical symptoms of hyperglycemia, even when DKA criteria were not met. No predefined biochemical threshold for hyperglycemia was required for this classification. In additional analyses, participants were divided in three groups: (i) non‐acute onset, (ii) acute onset without DKA and (iii) onset with DKA.

The primary endpoint variable was HbA1c. Secondary endpoints included body weight, Rx‐Risk, insulin doses, eGFR, and UACR and the development of chronic complications.

The index date, or baseline, was defined as the date of cohort entry, corresponding to the first visit from which systematic longitudinal clinical and laboratory data were available. Baseline variables therefore refer to cohort entry and not necessarily to the time of diabetes diagnosis. Endpoint variables were recorded at 3 months intervals up to 20 years. In a separate analysis, values of endpoint variables at each time point were fitted on a timeline starting at the date of T1D onset and were updated on a yearly basis up to 50 years after diagnosis. This complementary analysis placed available measurements on a diabetes‐duration time scale and assessed whether the findings were robust to differences in the interval between diagnosis and cohort entry.

### 2.4 Data Collection

We retrieved the following information at cohort entry, which did not necessarily correspond to the time of diagnosis: demographics (biological sex as assigned at birth; age at first visit, age at onset of T1D, duration of diabetes at first visit); anthropometrics (weight and height for the calculation of body mass index, BMI); systolic and diastolic blood pressure; laboratory exams (HbA1c, lipid profile, liver enzymes, urinary albumin excretion rate, serum creatinine for recalculating the estimated glomerular filtration rate [eGFR] according to the CKD‐EPI equation [15]); concomitant medications; use of technology (continuous subcutaneous insulin infusion [CSII]/insulin pumps and continuous glucose monitoring [CGM]). A comorbidity index was calculated based on remapping medications to their ATC class and using the Rx‐Risk equation [16].

We used the following definition of complications. A composite kidney endpoint was defined by an eGFR < 60 mL/min/1.73 m^2^ or UACR > 30 mg/g on at least one assessment; potential transient causes of albuminuria, such as urinary tract infection, exercise, menstruation, or acute illness, could not be systematically excluded from the retrospective dataset. Retinopathy was defined as the presence of diabetic retinopathy of any grade documented by digital funduscopic examination. Somatic neuropathy was defined based on the Michigan Neuropathy Score Index (MNSI), monofilament test and eventually confirmed by nerve conduction velocity; autonomic neuropathy was diagnosed in the presence of at least 2 altered cardiac autonomic tests (Valsalva manoeuver, deep breathing, lying to standing, orthostatic hypotension). Coronary artery disease was defined as myocardial infarction or angina, confirmed by imaging studies showing altered regional cardiac contractility (ultrasound) or at least 50% coronary artery stenosis (angiography). Cerebrovascular disease was defined as stroke or transient ischaemic attack, eventually confirmed by imaging with evidence of side‐coherent brain ischaemic lesion(s). Peripheral arterial disease was defined as an ankle‐brachial index (ABI) < 0.9 or evidence of arterial stenosis upon imaging studies (ultrasound or angiography). Asymptomatic atherosclerosis was defined as imaging evidence of carotid plaques with lumen narrowing of at least 30%. Microangiopathy was defined as the presence of either renal endpoint, retinopathy, or neuropathy. Macroangiopathy was defined as either CAD, PAD, cerebrovascular disease, or asymptomatic atherosclerosis. Cardiovascular disease was defined in the presence of symptomatic CAD, PAD or cerebrovascular disease. Screening for complications took place as part of routine clinical care, with frequency and protocols that could change over time.

### 2.5 Statistical Analysis

Data are presented as mean (standard deviation) for continuous variables or as number (percentage) for categorical variables. Non‐normal distributed variables were log‐transformed. Comparisons between the two groups were performed using unpaired 2‐tail Student’s *t* test for continuous variables and with chisquare test for categorical variables.

No missing data were imputed. Cross‐sectional descriptive and univariate analyses were performed using available data for each variable. Multivariable regression models were based on participants with available data for all variables included in the respective models. Mixed models for repeated measures used all available longitudinal observations and therefore did not require complete observations at every timepoint.

The trend over time in continuous outcome variables (e.g., HbA1c) was compared between groups using the mixed model for repeated measures (MMRM): the dependent variable was the change versus baseline in the outcome variable at each timepoint; time, group (e.g., with/without DKA at diagnosis), and time‐by‐group interaction were fixed factors; the initial value of the outcome variable (e.g., HbA1c) was included as a covariate to account for the dependence of subsequent change on the starting value; other covariates included features identified upon univariate between‐group comparisons. The first‐order autoregressive structure of covariance was chosen. The output of the MMRM included the mean (SE) value of the dependent variable in the two groups, the mean (SE) between‐group difference, and the marginal mean (SE) values at each time point used to draw graphs. In a separate analysis, we entered HbA1c values as the dependent variable, allowing to estimate the mean value in each group across the observation period.

To compare categorical outcomes between the two groups (e.g., complications), we used Cox regression models. Covariates included the belonging group (e.g., with/without DKA at diagnosis) and features identified upon univariate cross‐sectional comparison at baseline. Follow‐up was censored at the last available clinical contact at the study centre. Vital status after the last recorded visit was not systematically available. Hazard proportionality assumption was verified by visual inspection of Schoenfeld’s residuals. Complications documented at cohort entry were retained in the time‐to‐event analyses; when the exact date of occurrence after T1D diagnosis could not be reconstructed, the event was assigned a time of zero in analyses using cohort entry as the time origin. As a sensitivity analysis, models were refitted using diabetes duration as the underlying time scale, with delayed entry at cohort enrolment [17]. This approach accounted for differences in diabetes duration before cohort entry among the included participants but could not account for selection into the cohort before enrolment.

Statistical significance was set at the conventional type I error level of 5% without adjustment for multiple testing. SPSS ver. 30 was used.

## 3 Results

### 3.1 Study Participants

We included 517 individuals with adult‐onset T1D: 47.2% women, mean age at onset of 32.8 years. The first available outpatient visit in the database was recorded at a mean of 6.8 years after the onset of T1D. At baseline, in the entire cohort, BMI was 23.5 kg/m^2^ and HbA1c 8.4% (68 mmol/mol) (Table 1). All patients initiated therapy with multidose insulin.

**TABLE 1:** Baseline characteristics of study participants were divided according to the DKA at diagnosis.

Variable	All	Non‐DKA	DKA	*p*
Number	517	405	112	
Female, *n* (%)	244 (47.2)	198 (48.9)	46 (41.1)	0.142
Age at first visit, years	39.4 (12.8)	41.2 (12.6)	33.1 (11.8)	< 0.001
Age at onset, years	32.8 (12.3)	33.8 (12.5)	29.2 (10.6)	< 0.001
Disease duration at first visit, years	6.8 (9.8)	7.6 (10.0)	3.9 (8.6)	< 0.001
Weight, kg	69.6 (13.4)	70.0 (13.4)	68.5 (13.4)	0.228
Body mass index, kg/m^2^	23.5 (3.6)	23.6 (3.5)	23.0 (3.6)	0.021
Systolic blood pressure, mm Hg	124.6 (16.8)	125.6 (16.7)	120.9 (16.9)	0.005
Diastolic blood pressure, mm Hg	77.6 (10.2)	78.2 (10.2)	75.4 (10.0)	0.006
Laboratory				
HbA1c, %	8.4 (2.3)	8.2 (2.0)	9.1 (2.9)	< 0.001
mmol/mol	68.3 (25.1)	66.1 (21.9)	76.0 (31.7)	
Total cholesterol, mg/dl	185.3 (37.3)	187.6 (36.2)	176.9 (40.1)	0.005
HDL cholesterol, mg/dl	61.2 (17.4)	62.4 (17.0)	57.0 (18.3)	0.002
Triglycerides, mg/dl	88.7 (61.9)	87.3 (60.0)	93.9 (68.1)	0.486
LDL‐cholesterol, mg/dl	105.1 (30.3)	106.3 (29.6)	100.6 (32.6)	0.128
AST, IU/l	21.5 (7.5)	21.4 (7.3)	22.2 (8.4)	0.217
ALT, IU/l	23.8 (14.2)	23.5 (13.5)	24.9 (16.6)	0.170
Serum creatinine, mg/dl	0.8 (0.2)	0.8 (0.2)	0.8 (0.2)	0.975
eGFR, ml/min/1.73 m^2^	102.3 (17.7)	101.0 (16.8)	107.1 (20.1)	< 0.001
Urinary albumin/creatinine ratio, mg/g	16.9 (58.6)	16.2 (53.4)	19.6 (75.8)	0.914
Insulin pump use at cohort entry, *n* (%)	18 (3.5)	16 (4.0)	2 (1.8)	0.269

*Note:* Data recorded at time of cohort entry are presented as mean (standard deviation) or as number (percentage), when appropriate.

### 3.2 Presentation of T1D

Overall, non‐acute presentation was recorded in *n* = 176 participants (34.0%), whereas *n* = 229 and *n* = 112 developed T1D with acute non‐DKA presentation (44.3%) and DKA (21.7%), respectively.

Table 1 compares clinical characteristics of participants with or without DKA at diagnosis. DKA was associated with younger age at onset, shorter disease duration at cohort entry (3.9 vs. 7.6 years), higher HbA1c and eGFR, slightly lower BMI, cholesterol and blood pressure. Comparisons between acute versus non‐acute (both DKA and non‐DKA) and across the three groups are shown in the Supporting Information S1: Table S1 and S2 of the Appendix.

### 3.3 Trends in T1D Presentation

We divided the data collection period into quartiles and examined the trend of onset modality (Figure 1). The distribution of presentation patterns changed significantly over time (χ^2^ *p* < 0.001). The proportion of patients presenting with DKA increased from 13.2% in 2000–2008 to 34.6% after 2016. A significant positive temporal trend in DKA frequency was observed (Cochran–Armitage trend test, *p* < 0.001). Increasing calendar year of T1D onset remained significantly associated with a higher frequency of DKA presentation after adjustment for confounders. The other independent factors were younger age at onset and higher values of the first recorded HbA1c at cohort entry (Supporting Information S1: Table S3 of the Appendix).

**FIGURE 1:**
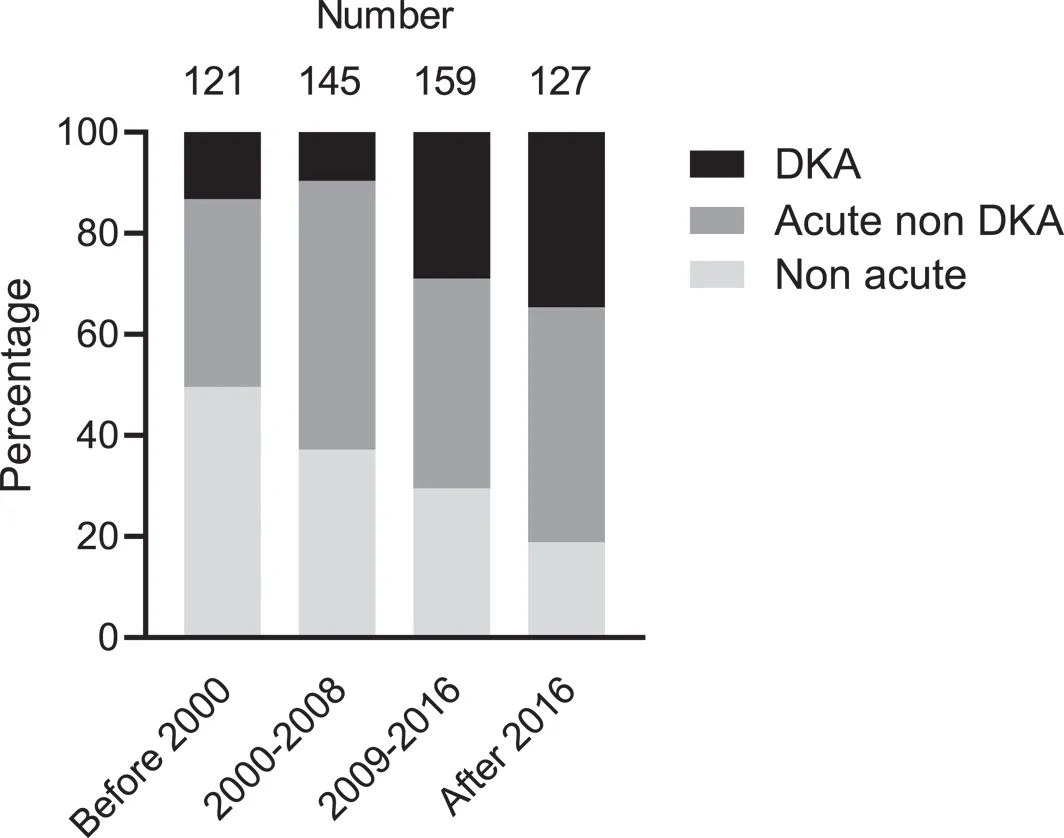
Modality of T1D diagnosis according to study period. The study period was divided into quartiles and the modality of presentation was reported in each period. Statistics are provided in Table S3.

### 3.4 Presentation With DKA and Long‐Term Glycaemic Control

We examined the long‐term change in HbA1c according to the presence or absence of DKA at diagnosis (Figure 2). The MMRM was adjusted for covariates identified in the between‐group comparison, including HbA1c at cohort entry as the baseline value of the outcome and age at onset, disease duration at cohort entry, sex, BMI, systolic blood pressure, total cholesterol, and eGFR. The observation period spanned up to 20 years after cohort entry (median 9.3 years, IQR 3.5–15.5 years). Follow‐up was shorter in the DKA group (median 7.1 vs. 10.9 years) consistently with the increasing prevalence of DKA at onset over time. There were on average 31.1 HbA1c values/patient in the non‐DKA group versus 24.6 values/patient in the DKA group (*p* < 0.001). HbA1c declined by 0.82% (9.0 mmol/mol) in the non‐DKA group and by 0.45% (4.9 mmol/mol) in the DKA group, with an estimated difference of 0.37% (4.0 mmol/mol; 95% C.I. 0.20%–0.53% or 2.2–5.8 mmol/mol; *p* < 0.001; Figure 2A,B).

**FIGURE 2:**
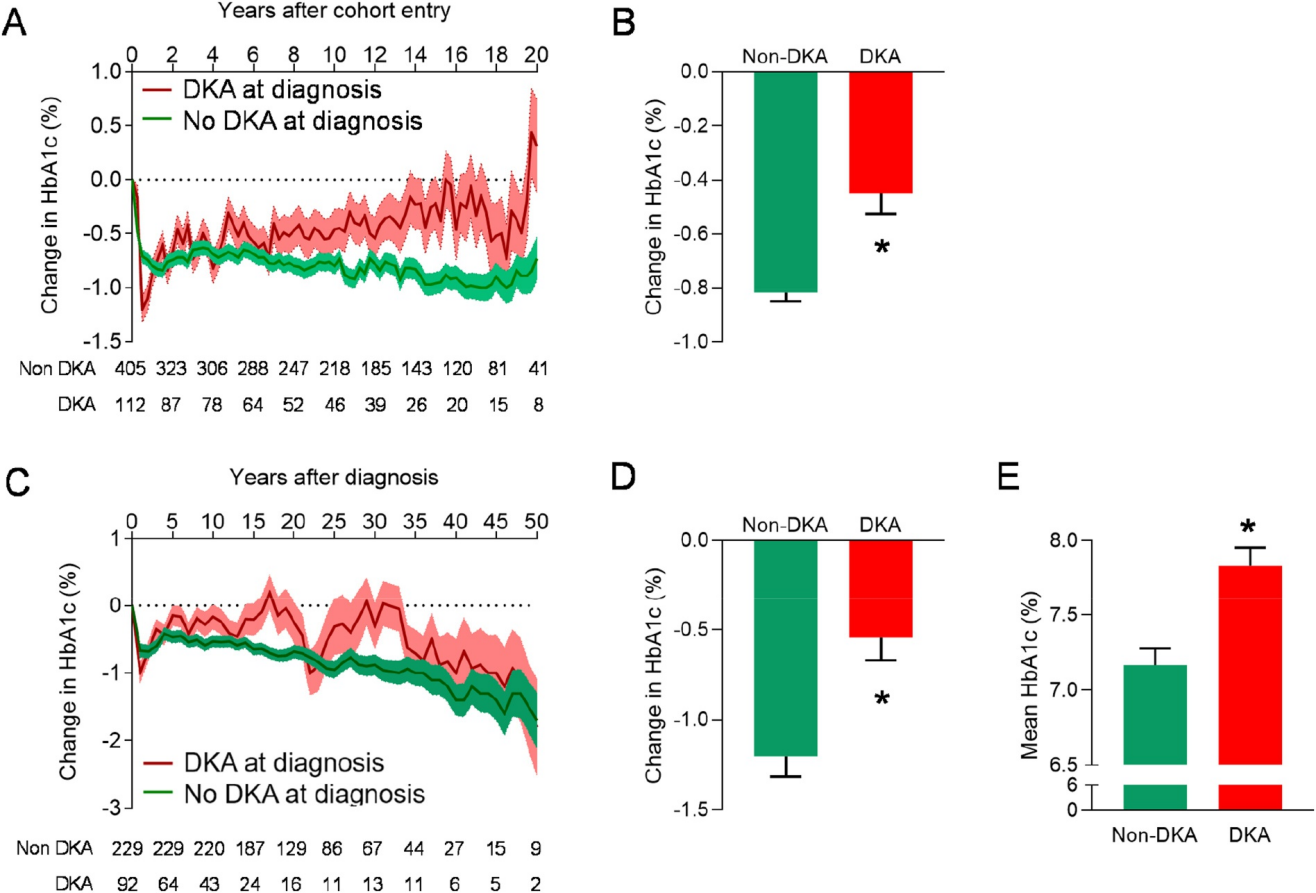
DKA at diagnosis and glycaemic trajectories. (A) Change in HbA1c from cohort entry in participants with T1D and with/without DKA at diagnosis. (B) Mean (SE) estimated change in HbA1c during the period observed in panel A. (C) Change in HbA1c from time of diagnosis in participants with T1D and with/without DKA at diagnosis. (D) Mean (SE) estimated change in HbA1c during the period observed in panel C. (E) Mean values of HbA1c during the period observed in panel C in the two groups. The tables below the plots in panels A and C report the number of unique participants contributing at least one HbA1c measurement within each time interval. *p < 0.05.

When data were fitted on a timeline starting from diagnosis and spanning up to 50 years, the estimated mean between‐group difference was 0.66% (7.2 mmol/mol; *p* < 0.001; Figure 2C,D). The overall HbA1c during the period was 7.17% (54.9 mmol/mol) in the non‐DKA group and 7.83% (62.1 mmol/mol) in the DKA group (*p* < 0.001 for difference; Figure 2E).

C‐peptide concentrations were available for a minority of participants (*n* = 165) at heterogeneous timepoints during follow‐up. The proportion of participants with clinically relevant residual beta‐cell function (> 0.60 ng/ml) did not differ significantly between those with and without DKA at diagnosis (52.4% vs. 60.4%, respectively), but these data are unlikely to be representative. Insulin dose/kg of body weight remained similar in the two groups throughout observation (0.4 U/kg) and no difference in the change in body weight was observed (not shown).

Of note, participants presenting with DKA were less likely to transition to CSII therapy during follow‐up (21.4% vs. 39.0%), independently of age at onset, sex, diabetes duration at cohort entry, and calendar year of diagnosis (OR 0.39; 95% CI 0.23–0.68; *p* < 0.001). On the other hand, the proportion of those using CGM was very similar in the two groups (91.1% vs. 90.6%).

### 3.5 Presentation With DKA and Complications

To evaluate the association between presentation with DKA and incident complications, we used Cox regression models adjusted for the same confounders used in MMRM. Retinopathy was documented in 111 participants without DKA and 29 participants with DKA. Participants with T1D who were diagnosed with DKA had a significantly higher incidence of retinopathy than those without DKA presentation (adjusted HR 1.61; 95% C.I. 1.02–2.55; *p* = 0.041; Figure 3). The association between DKA at diagnosis and incident retinopathy was unchanged after additional adjustment for the calendar year of T1D diagnosis (HR 1.81; 95% CI 1.14–2.86). Because 10 participants entered the cohort with prevalent retinopathy (6 with DKA at onset and 4 without) in a sensitivity analysis, we refitted Cox regression models using diabetes duration as the time scale and accounting for delayed entry at cohort enrolment. The association between DKA at diagnosis of T1D and incident retinopathy remained unchanged, with a 66% relative higher rate of retinopathy among participants presenting with DKA (HR 1.66; 95% CI 1.07–2.58; *p* = 0.024).

**FIGURE 3:**
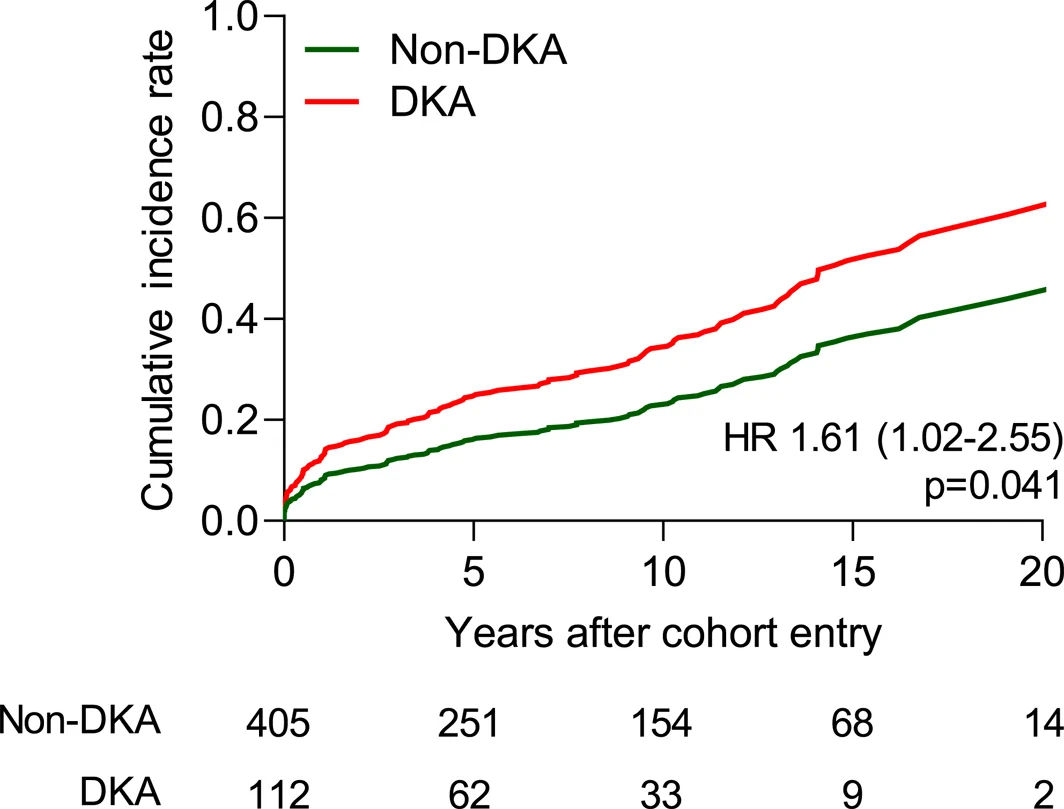
Model‐based cumulative incidence curves of retinopathy. Data are derived from adjusted Cox proportional hazard models. The table below the plot reports the number of participants at risk at the various timepoints in each group.

In an exploratory analysis, adjustment for mean HbA1c during follow‐up attenuated the association between DKA and incident retinopathy (HR 1.07; 95% CI 0.64–1.78; *p* = 0.794), with mean HbA1c remaining independently associated with retinopathy risk (HR 1.61; 95% CI 1.37–1.88; *p* < 0.001; Supporting Information S1: Table S4 in the Appendix).

For other complications examined, HR was > 1.0 for DKA presentation but was non‐statistically significant (Supporting Information S1: Table S5 in the Appendix). We also found no differences in the changes over time in eGFR and UACR (Supporting Information S1: Figure S1 in the Appendix). The comorbidity risk score based on medications showed similar trends in the two groups (not shown).

### 3.6 Acute Onset and Long‐Term Outcomes

We then repeated the analysis comparing acute T1D onset (including DKA and non‐DKA) versus non‐acute onset (Supporting Information S1: Table S2). Participants with acute onset had younger age and shorter diabetes duration at cohort entry and higher HbA1c and eGFR. They displayed a different trend of HbA1c over time (time‐by‐group interaction term *p* = 0.003), with greater improvement in the first 2 years, followed by stabilisation and a trend towards long‐term higher values compared to participants with non‐acute onset (Supporting Information S1: Figure S2), although the mean change was similar in the two groups: −0.73% (−8.0 mmol/mol) versus −0.77% (−8.4 mmol/mol); *p* = 0.505.

Participants with acute onset had a significantly higher rate of incident retinopathy (HR 1.53; 95% C.I. 1.05–2.24; *p* = 0.028; Supporting Information S1: Table S5), but no difference in other complications and similar changes over time in eGFR and UACR (not shown).

## 4 Discussion

In this retrospective cohort of people with adult‐onset T1D, presentation with DKA was associated with persistently worse glycaemic control and a higher incidence of retinopathy during long‐term follow‐up.

The frequency of DKA at diagnosis increased substantially over the study period. This is consistent with reports from paediatric populations showing persistently high or increasing rates of DKA at T1D diagnosis [3, 18]. Possible explanations for this trend include delayed recognition of hyperglycaemic symptoms, increasing diagnostic complexity of adult‐onset autoimmune diabetes, and broader healthcare‐system and societal factors affecting access to timely diagnosis [6, 8, 19]. In addition, disruptions in healthcare access related to the COVID‐19 pandemic may have contributed to the patterns observed in most recent years [20]. Although recall bias and changes in referral patterns or case mix over the long study period cannot be excluded, the observed trend remained significant after adjustment for confounders. Therefore, our findings extend observations from children to adults, highlighting that DKA at diagnosis remains a relevant clinical and public health challenge.

In paediatric cohorts, DKA at diagnosis has been associated with lower residual beta‐cell function and poorer metabolic control during follow‐up [4, 5, 21, 22]. The most important finding of the present study is that DKA at diagnosis was associated with less favourable long‐term glycaemic control from two complementary perspectives. In the change‐from‐baseline analysis, participants presenting with DKA showed a smaller reduction in HbA1c from cohort entry, whereas the analysis of absolute HbA1c values showed that they maintained higher HbA1c levels for decades after diagnosis. Such a persistent separation in glycaemic trajectories is unlikely to be explained solely by the acute metabolic disturbance occurring at diagnosis and suggests that DKA may identify individuals with distinct disease characteristics already present at onset.

DKA at diagnosis should therefore be interpreted primarily as a clinical marker associated with subsequent outcomes rather than as an independent causal effect. Several mechanisms may underlie this association. DKA at diagnosis may reflect a more aggressive autoimmune process leading to more extensive beta‐cell destruction and lower residual endogenous insulin secretion. DKA may represent a marker of delayed disease recognition, lower diabetes awareness or health literacy, or adverse social and healthcare determinants that continue to influence disease management long after diagnosis. The fact that fewer HbA1c values were available for participants in the DKA group may suggest a lower number of office consultations and a less stringent follow‐up. Although the MMRM used all available longitudinal observations and did not require complete measurements at each timepoint, differential frequency of HbA1c assessment between groups could still have influenced the estimated trajectories if the observation pattern was related to glycaemic control.

Interestingly, participants presenting with DKA were less likely to transition to pump therapy during follow‐up, even after adjustment for major demographic and temporal factors. Rather than implying a direct effect of DKA on treatment allocation, this finding may reflect differences in access to diabetes technologies, treatment preferences, healthcare engagement, or unmeasured socioeconomic and healthcare‐related factors, which may in turn contribute to the observed divergence in long‐term glycaemic control. These mechanisms are not mutually exclusive and may jointly contribute to the observed differences in long‐term glycaemic control.

In addition to worse metabolic outcomes, participants presenting with DKA experienced a significantly higher incidence of retinopathy. Although the magnitude of the association was moderate, it remained consistent in sensitivity analyses accounting for delayed cohort entry and differences in diabetes duration. Retinopathy was the only complication significantly associated with DKA at diagnosis and the small number of participants remaining at risk at later time points warrants caution in interpreting the late portion of the curves, suggesting that this finding should be considered exploratory and requires confirmation. The association is nonetheless biologically plausible given the well‐established relationship between cumulative glycaemic exposure and retinopathy [23, 24]. When mean HbA1c during follow‐up was added to the model, the association between DKA and retinopathy was attenuated. Although this exploratory analysis should not be interpreted as formal mediation, it suggests that greater glycaemic exposure contributed to the higher retinopathy risk observed in participants presenting with DKA. The less frequent access to pump therapy in the DKA group may also have contributed to higher HbA1c and greater retinopathy risk, as pump therapy has been associated with reduced rates of retinopathy progression [25].

The absence of a detectable association between DKA at diagnosis and kidney outcomes deserves specific consideration. Although diabetic kidney disease and retinopathy share glucose‐mediated pathogenic pathways [26], kidney outcomes are influenced by a broader range of factors, including blood pressure, smoking, obesity, and pharmacological interventions [27, 28, 29]. In our cohort, average HbA1c levels remained below 8% even among participants presenting with DKA: at such levels of glycaemic exposure, non‐glycaemic determinants of kidney disease may outweigh the contribution of hyperglycemia.

The comparison between acute and non‐acute disease presentation provides additional insight into the specificity of the observed associations. Although acute presentation was associated with a higher incidence of retinopathy, differences in long‐term glycaemic control were substantially less pronounced than those observed for DKA. These findings suggest that the prognostic signal identified in our study may be more specifically related to the presence of ketoacidosis at diagnosis.

Our findings have potential clinical implications: presentation with DKA could be part of stratification strategies to identify adults who may benefit from closer metabolic surveillance, earlier adoption of diabetes technologies, or intensified educational interventions. Furthermore, the observation that DKA may have implications extending far beyond the acute phase reinforces the importance of strategies aimed at preventing DKA at disease onset [11, 12, 18]. In this context, increasing efforts towards earlier recognition of T1D and population‐based screening may provide benefits beyond reducing acute morbidity [30, 31, 32]. Early identification of stage I‐II T1D in adults could substantially decrease the risk of DKA at diagnosis and may facilitate access to disease‐modifying therapies that delay progression to stage III [33, 34].

The strengths of this study include the relatively large cohort of adults with T1D, the detailed phenotypic characterisation of disease presentation, the long duration of follow‐up, and the availability of repeated longitudinal measurements allowing the reconstruction of long‐term glycaemic trajectories and complication rates.

Several limitations should be acknowledged. First, the study was retrospective and conducted at a single tertiary centre, which may limit the generalizability of the findings. Second, information on DKA at diagnosis was reconstructed from historical clinical records and patient interviews and may have been subject to misclassification. Third, C‐peptide data were available for a minority of participants and were not collected systematically, making them highly susceptible to ascertainment bias. Therefore, we could not reliably assess the contribution of residual beta‐cell function to the association between DKA at diagnosis and long‐term outcomes. HbA1c values were collected from multiple laboratories and not always IFCC‐aligned, especially in the early years. Fourth, data on socioeconomic and social determinants of health were unavailable. Such factors may influence both the likelihood of presenting with DKA and subsequent diabetes management [6, 35], and residual confounding by unmeasured social and healthcare‐related variables cannot be excluded. For retinopathy, we acknowledge that retinal screening was performed according to routine clinical practice without standardised timing and frequency, and grading systems evolved over the long study period. Vital status after the last available clinical contact was not systematically available; therefore, death could not be distinguished from other reasons for discontinuation of follow‐up and could not be modelled as a competing event. Informative censoring therefore cannot be excluded. Finally, cohort entry occurred years after T1D diagnosis and earlier in participants presenting with DKA. Analyses using diabetes duration as the time scale with delayed entry addressed differences in disease duration at the time of cohort entry but cannot eliminate potential selection related to survival, referral patterns, healthcare access, or attendance at a tertiary diabetes clinic before cohort entry. The study therefore does not represent an inception cohort of adults with newly diagnosed T1D, and these factors may limit the generalizability of the observed associations.

In conclusion, among adults with T1D attending a tertiary diabetes clinic, DKA at diagnosis was associated with persistently less favourable glycaemic control and, in exploratory analyses, a higher incidence of retinopathy during long‐term follow‐up. Future studies should investigate the biological, behavioural, and social mechanisms underlying these associations and determine whether prevention of DKA at diagnosis translates into improved long‐term outcomes. In the era of population‐based screening for pre‐symptomatic T1D and emerging disease‐modifying immunotherapies, preventing DKA at disease onset remains a major goal of care.

## Author Contributions

Data research and analysis: R.M.P, F.B, M.L.M, G.P.F. Data collection and interpretation: D.F, C.B, M.C.C, M.V, D.B, A.M, B.M.B. Manuscript writing R.M.P, G.P.F. Manuscript revision: F.B, M.L.M. All authors contributed substantially to the study conception and design and/or data acquisition, analysis, and interpretation. All authors participated in drafting or critically revising the manuscript, approved the final version, and agree to be accountable for all aspects of the work.

## Funding

This work was supported by Università degli Studi di Padova.

## Conflicts of Interest

R.M.P. received honoraria, consultancy or lecture fees from Astrazeneca, Novo Nordisk, Sanofi. F.B. received grants, honoraria or lecture fees from Novo Nordisk, Roche, Sanofi Aventis, Glooko, Tandem, Movi. D.B. received congress invitations, honoraria and consultancy fees from Abbott, Eli Lilly, Menarini, Novo Nordisk, Roche Diabetes Care, Sanofi, Terumo, Theras and Ypsomed and served on advisory board panels for Roche Diabetes Care and Terumo. A.M. received lecture fees from Novo Nordisk. B.M.B. received lecture or advisory board fees from AstraZeneca, Boehringer Ingelheim, Lilly, M.S.D., Sanofi, Servier and Novo Nordisk. M.L.M. received honoraria or speaker fees from Amgen, AstraZeneca, Boehringer, Daiichi Sankyo, Lilly, Guidotti, Merck Sharp & Dohme, Novartis, Novo Nordisk, Sanofi, and Servier. G.P.F. received honoraria or lecture fees from Abbott, Astra Zeneca, Boehringer, Daichii‐Sankyo, Guidotti, Lilly, Merck Sharp & Dohme, Novo Nordisk, Novartis, Sanofi. D.F., C.B., M.C.C., M.V. have nothing to disclose.

## Supporting information


Supporting Information S1

## Data Availability

Research data are not shared.
