# Supplementary material for: Diabetic Ketoacidosis at Diagnosis is Associated With Adverse Long‐Term Glycaemic Trajectories and Retinopathy in Adult‐Onset Type 1 Diabetes. A Single‐Centre Retrospective Cohort Study
Source: Diabetes Metab Res Rev. 2026 Nov 3;42(8):e70249. doi: 10.1002/dmrr.70249 (PMC13729818; doi:10.1002/dmrr.70249)
Supplement: Supplementary file 1 [file DMRR-42-e70249-s001.docx]

**Diabetic ketoacidosis at diagnosis is associated with adverse long-term glycemic trajectories and retinopathy in adult-onset type 1 diabetes.**

**A single-center retrospective cohort study**

Pollis et al.

**Appendix**

| **Table of contents** | **Page** |
| --- | --- |
| Table S1. Clinical characteristics of study participants divided by modality of presentation | 2 |
| Table S2. Clinical characteristics of study participants divided according to acute and non-acute presentation | 3 |
| Table S3. Factors independently associated with DKA at diagnosis | 4 |
| Table S4. Cox proportional hazard models for retinopathy incidence | 5 |
| Table S5. Modality of onset and complications | 6 |
| Figure S1. Change over time in markers of kidney function and damage according to modality of presentation | 7 |
| Figure S2. Change from cohort entry in HbA1c in participants with acute versus non-acute presentation of T1D | 8 |

**Table S1. Clinical characteristics of study participants divided by modality of presentation**. Data are presented as mean (standard deviation) or as number (percentage), when appropriate. p1 refers to the p-value of the comparison between acute vs non-acute, whereas p2 refers to the p-value of the comparison between DKA vs non-acute.

|  | **All** | **Non-acute** | **Acute non-DKA** | **DKA** | **p1** | **p2** |
| --- | --- | --- | --- | --- | --- | --- |
| Number | 517 | 176 | 229 | 112 |  |  |
| Female, n (%) | 244 (47.2) | 90 (51.1) | 108 (47.2) | 46 (41.1) | 0.429 | 0.096 |
| Age at first visit, years | 39.4 (12.8) | 43.6 (12.4) | 39.3 (12.4) | 33.1 (11.8) | <0.001 | <0.001 |
| Age at onset, years | 32.8 (12.3) | 34.5 (12.6) | 33.3 (12.5) | 29.2 (10.6) | 0.368 | <0.001 |
| Disease duration at first visit, years | 6.8 (9.8) | 9.6 (11.3) | 6.0 (8.5) | 3.9 (8.6) | <0.001 | <0.001 |
| Weight, kg | 69.6 (13.4) | 70.4 (12.5) | 69.6 (14.0) | 68.5 (13.4) | 0.545 | 0.228 |
| Body mass index, kg/m2 | 23.5 (3.6) | 23.9 (3.3) | 23.4 (3.7) | 23.0 (3.6) | 0.113 | 0.021 |
| Systolic blood pressure, mm Hg | 124.6 (16.8) | 126.7 (16.2) | 124.8 (17.1) | 120.9 (16.9) | 0.270 | 0.005 |
| Diastolic blood pressure, mm Hg | 77.6 (10.2) | 78.8 (9.9) | 77.8 (10.5) | 75.4 (10.0) | 0.368 | 0.006 |
| Laboratory |  |  |  |  |  |  |
| HbA1c, %  mmol/mol | 8.4 (2.3)  68 (25) | 7.8 (1.5)  62 (16) | 8.6 (2.3)  70 (25) | 9.1 (2.9)  76 (32) | <0.001 | <0.001 |
| Total cholesterol, mg/dl | 185.3 (37.3) | 189.5 (34.3) | 186.2 (37.8) | 176.9 (40.1) | 0.371 | 0.005 |
| HDL cholesterol, mg/dl | 61.2 (17.4) | 63.9 (17.9) | 61.1 (16.3) | 57.0 (18.3) | 0.099 | 0.002 |
| Triglycerides, mg/dl | 88.7 (61.9) | 88.7 (55.8) | 86.2 (63.3) | 93.9 (68.1) | 0.685 | 0.486 |
| LDL-cholesterol, mg/dl | 105.1 (30.3) | 106.2 (27.6) | 106.4 (31.1) | 100.6 (32.6) | 0.948 | 0.128 |
| AST, IU/l | 21.5 (7.5) | 21.1 (6.7) | 21.5 (7.8) | 22.2 (8.4) | 0.552 | 0.217 |
| ALT, IU/l | 23.8 (14.2) | 22.6 (11.9) | 24.2 (14.6) | 24.9 (16.6) | 0.250 | 0.170 |
| Serum creatinine, mg/dl | 0.8 (0.2) | 0.8 (0.2) | 0.8 (0.2) | 0.8 (0.2) | 0.157 | 0.975 |
| eGFR, ml/min/1.73 m2 | 102.3 (17.7) | 97.7 (16.2) | 103.6 (16.9) | 107.1 (20.1) | <0.001 | <0.001 |
| UACR, mg/g | 16.9 (58.6) | 20.6 (62.9) | 12.7 (44.2) | 19.6 (75.8) | 0.153 | 0.914 |

**Table S2. Clinical characteristics of study participants divided according to acute and non-acute presentation**. Data are presented as mean (standard deviation) or as number (percentage), when appropriate.

|  | All | Non-acute | Acute | p |
| --- | --- | --- | --- | --- |
| Number | 517 | 176 | 341 |  |
| Female, n (%) | 244 (47.2) | 90 (51.1) | 154 (45.2) | 0.429 |
| Age at first visit, years | 39.4 (12.8) | 43.6 (12.4) | 37.3 (12.6) | <0.001 |
| Age at onset, years | 32.8 (12.3) | 34.5 (12.6) | 32.0 (12.1) | 0.368 |
| Disease duration at first visit, years | 6.8 (9.8) | 9.6 (11.3) | 5.3 (8.6) | <0.001 |
| Weight, kg | 69.6 (13.4) | 70.4 (12.5) | 69.2 (13.8) | 0.545 |
| Body mass index, kg/m2 | 23.5 (3.6) | 23.9 (3.3) | 23.2 (3.6) | 0.113 |
| Systolic blood pressure, mm Hg | 124.6 (16.8) | 126.7 (16.2) | 123.6 (17.1) | 0.270 |
| Diastolic blood pressure, mm Hg | 77.6 (10.2) | 78.8 (9.9) | 77.0 (10.4) | 0.368 |
| Laboratory |  |  |  |  |
| HbA1c, % | 8.4 (2.3) | 7.8 (1.5) | 8.8 (2.5) | <0.001 |
| Total cholesterol, mg/dl | 185.3 (37.3) | 189.5 (34.3) | 183.1 (38.7) | 0.371 |
| HDL cholesterol, mg/dl | 61.2 (17.4) | 63.9 (17.9) | 59.7 (17.1) | 0.099 |
| Triglycerides, mg/dl | 88.7 (61.9) | 88.7 (55.8) | 88.8 (64.9) | 0.685 |
| LDL-cholesterol, mg/dl | 105.1 (30.3) | 106.2 (27.6) | 104.5 (31.7) | 0.948 |
| AST, IU/l | 21.5 (7.5) | 21.1 (6.7) | 21.8 (8.0) | 0.552 |
| ALT, IU/l | 23.8 (14.2) | 22.6 (11.9) | 24.4 (15.2) | 0.250 |
| Serum creatinine, mg/dl | 0.8 (0.2) | 0.8 (0.2) | 0.8 (0.2) | 0.157 |
| eGFR, ml/min/1.73 m2 | 102.3 (17.7) | 97.7 (16.2) | 104.7 (18.0) | <0.001 |
| UACR, mg/g | 16.9 (58.6) | 20.6 (62.9) | 14.9 (56.2) | 0.153 |
| TSH, IU/l | 2.2 (1.7) | 2.0 (1.2) | 2.3 (1.8) | 0.059 |

**Table S3. Factors independently associated with DKA presentation**. A logistic regression model was run with DKA presentation as the dependent variable and covariates entered as a block.

| **Variable** | **Odds ratio (95% C.I.)** | **p-value** |
| --- | --- | --- |
| Calendar year of T1D onset | 1.04 (1.00-1.08) | 0.034 |
| Female sex | 0.81 (0.51-1.29) | 0.376 |
| Age at onset, years | 0.95 (0.93-0.97) | <0.001 |
| Disease duration at cohort entry, years | 0.99 (0.93-1.04) | 0.591 |
| Body mass index, kg/m^2^ | 0.99 (0.93-1.06) | 0.855 |
| First recorded HbA1c, % | 1.14 (1.05-1.25) | 0.004 |

**Table S4. Cox proportional hazard models for retinopathy incidence**. Model 1 was run entering covariates with the first HbA1c recorded at cohort entry. Model 2 was run including also the mean HbA1c recorded during follow-up but before retinopathy incidence.

|  | **Model 1** | | **Model 2** | |
| --- | --- | --- | --- | --- |
| **Variable** | HR (95% CI) | p-value | HR (95% CI) | p-value |
| DKA at diagnosis | 1.61 (1.02-2.55) | 0.041 | 1.07 (0.64-1.78) | 0.794 |
| Female sex | 0.72 (0.51-1.04) | 0.077 | 0.64 (0.43-0.95) | 0.025 |
| Age at onset | 1.01 (0.99-1.03) | 0.385 | 1.01 (0.99-1.03) | 0.438 |
| Disease duration at cohort entry | 1.06 (1.04-1.08) | <0.001 | 1.06 (1.04-1.08) | <0.001 |
| Body mass index | 1.01 (0.96-1.06) | 0.826 | 0.98 (0.93-1.04) | 0.533 |
| Systolic blood pressure | 1.01 (0.99-1.02) | 0.140 | 1.00 (0.99-1.02) | 0.658 |
| Total cholesterol | 0.99 (0.99-1.01) | 0.092 | 0.99 (0.99-0.99) | 0.027 |
| eGFR | 0.99 (0.98-1.01) | 0.225 | 1.00 (0.99-1.01) | 0.720 |
| First HbA1c at cohort entry | 1.15 (1.06-1.25) | <0.001 | 1.02 (0.91-1.14) | 0.787 |
| Mean HbA1c in the entire period | - | - | 1.61 (1.37-1.88) | <0.001 |

**Table S5. Modality of onset and complications**. For DKA versus non-DKA and for acute versus non-acute presentation, we ran separate Cox proportional hazards models for the various complications, adjusted for variables selected from the univariate comparisons between groups.

| **Complication** | **DKA vs Non-DKA** | | **Acute vs Non-acute** | |
| --- | --- | --- | --- | --- |
|  | HR (95% C.I.) | p-value | HR (95% C.I.) | p-value |
| Retinopathy | 1.61 (1.02-2.55) | 0.041 | 1.53 (1.05-2.24) | 0.028 |
| Microangiopathy | 1.23 (0.84-1.81) | 0.287 | 1.17 (0.87-1.56) | 0.305 |
| Macroangiopathy | 1.29 (0.71-2.33) | 0.409 | 1.00 (0.63-1.58) | 1.000 |
| CAD | 1.25 (0.62-2.54) | 0.536 | 0.75 (0.43-1.30) | 0.302 |
| Composite kidney endpoint | 1.36 (0.85-2.18) | 0.198 | 1.15 (0.79-1.68) | 0.467 |

Model for DKA at diagnosis adjusted for sex, age at onset, duration at first visit, BMI, SBP, total cholesterol, eGFR, HbA1c.

Model for acute onset adjusted for sex, age, duration at first visit, eGFR, HbA1c.

The composite kidney endpoint was defined as eGFR <60 mL/min/1.73 m² or UACR >30 mg/g on at least one assessment.

**Figure S1. Change over time in markers of kidney function and damage according to modality of presentation**. Participants were divided according to the presence or absence of DKA at diagnosis of T1D. Panel A shows the mean change from cohort entry in eGFR, whereas panel B shows the change in urinary albumin excretion ratio (UACR) as the ratio of geometric mean.

**Figure S2. Change from cohort entry in HbA1c in participants with acute versus non-acute presentation of T1D**. In this analysis performed with the mixed model for repeated measures, the interaction term between time and group was statistically significant (p=0.003), although the mean effect was similar in the two groups (-0.77% in the non-acute group and -0.73% in the acute group).
